# Study on Pharmacological Treatment of Impulse Control Disorders in Parkinson’s Disease

**DOI:** 10.3390/jcm13226708

**Published:** 2024-11-08

**Authors:** Emilia Furdu-Lunguț, Claudia Antal, Suzana Turcu, Dan-Gabriel Costea, Mihai Mitran, Loredana Mitran, Andrei-Sebastian Diaconescu, Marius-Bogdan Novac, Gabriel-Petre Gorecki

**Affiliations:** 1 Department of Neurology, Faculty of Medicine, “Titu Maiorescu” University, 031593 Bucharest, Romania; emilia.furdu@spcf2.ro; 2 Department of Neurology, CF2 Clinical Hospital, 011464 Bucharest, Romania; 3 2gether NHS Foundation Trust for Gloucestershire, Gloucestershire GL3 4AW, UK; claudiaantal@yahoo.com; 4 “Francisc I. Rainer” Institute of Anthropology, Romanian Academy, 050711 Bucharest, Romania; suzana.turcu@antropologia.ro; 5 Department of General Surgery, CF2 Clinical Hospital, 011464 Bucharest, Romania; dancostea2006@yahoo.com; 6 Department of Obstetrics and Gynecology, Carol Davila University of Medicine and Farmacy, 050474 Bucharest, Romania; mihai.mitran@umfcd.ro; 7 Department of Obstetrics and Gynecology, “Prof. Dr. Panait Sîrbu” Clinical Hospital, 060251 Bucharest, Romania; 8 Department of ENT, Elias University Emergency Hospital, 011461 Bucharest, Romania; loredana.mitran@spitalul-elias.ro; 9 Department of General Surgery, Carol Davila University of Medicine and Farmacy, 050474 Bucharest, Romania; diaconescu_andrei@yahoo.com; 10 Department of General Surgery, Fundeni Clinical Institute, 022328 Bucharest, Romania; 11 Department of Anesthesiology and Intensive Care, University of Medicine and Farmacy Craiova, 200349 Craiova, Romania; mariusnovac2005@yahoo.com; 12 Department of Anesthesia and Intensive Care, Faculty of Medicine, “Titu Maiorescu” University, 031593 Bucharest, Romania; 13 Department of Anesthesia and Intensive Care, CF2 Clinical Hospital, 011464 Bucharest, Romania

**Keywords:** pharmacological treatment, Parkinson disease, impulsive control disorders, applied statistics

## Abstract

Parkinson’s disease is neurodegenerative, and additionally, a percentage higher than 60% is represented by the patients with associated psychiatric symptoms, such as anxiety disorders and depression. Due to illness itself and to therapy secondary effects, there is a high risk for these patients to develop impulse control disorders like ICDs: compulsive shopping, pathological gambling binge eating disorder, and hypersexuality. There is high interest in therapy so as to diminish, as much as possible, the associated ICD symptoms. This article presents a study on pharmacological treatment of impulse control disorders in Parkinson disease carried on a sample of patients in hospitals where the authors have their clinical work. This study was carried on for a period of 6 years and is focused on research of different treatment plans. The patients were evaluated by the Hamilton Rating Scale. Statistical analysis of the obtained data (given by the HAM-A scores) is used for data processing. All patients showed a reduction in their impulse behavior. The directions of further research development are mentioned.

## 1. Introduction

Nowadays, the reality of statistical data ranks Parkinson’s disease (PD) as the second neurodegenerative disease after Alzheimer’s [1]. Clinically, PD is characterized by resting tremors with a frequency of 4–7 cycles per second, muscular rigidity, and, therefore, postural instability. Additionally, there would be present bradykinesia physical signs. The motor phenotype is caused by dopamine deficiency in the basal ganglia because of the degeneration of dopaminergic neurons in the brain substantia nigra pars compacta. The Lewy bodies that represent the accumulated intracytoplasmic inclusion are present in surviving neurons when cell death is imminent.

Patients diagnosed with PD do exhibit a wide spectrum of non-motor symptoms, for example, sleep disturbances, neurobehavioral disorders, intellectual disability, apathy, dysphonia, neurobehavioral disorders, autonomic dysfunctions, and sleep disturbances. All these symptoms stand as background for the assumption that different parts of the nervous system are to be considered responsible for the disease, too, pointing towards neurotransmitters different than dopamine.

The International Parkinson and Movement Disorder Society (MDS) indicated a set of criteria aimed at achieving high accuracy in the diagnosis of Parkinson’s disease [2]. It is mentioned as a fundamental criterion of Parkinsonism, meaning the bradykinesia to which is added the rest tremor and/or the rigidity.

As mentioned in [3], there are approximately 1.2 million patients that have been diagnosed with Parkinson’s disease, and there is an estimated exponential increase in their number for the next decades. Great attention is given to the Body-Worn Sensors (BWS), meaning the patient’s wearable devices used for monitoring this disease’s clinical features like tremors (frequency and/or amplitude), motor fluctuations, dyskinesia, etc.

A detailed study presented in [4] evidences the encouraging research results in the development of a new method for evaluating the specific parameters (amplitude and constancy) of resting tremor in patients with Parkinson’s disease. This is based on the data obtained from tri-axial accelerometer components of patients’ smartwatches and on the hierarchical approach developed for patient tremor evaluation.

Of the patients with Parkinson’s disease, a percentage greater than 60% is represented by the ones having associated psychiatric symptoms, such as anxiety disorders, depression, and impulse control disorders [5]. These symptoms commonly dominate the clinical picture in severe PD and have a significant impact on quality of life and disability [6,7,8,9].

As far as the present knowledge is in Parkinson’s disease, no really efficient curative treatment could be recommended. The therapeutic strategies are based on a symptomatic approach focused on diminishing the dopaminergic deficit and providing symptomatic relief by dopamine replacement with levodopa, dopamine agonists, MAO-B inhibitors (monoamine oxidase B), and deep brain stimulation [10].

The therapy based on dopamine replacement is targeted toward the level of the depleted dorsal striatum and of the relatively intact ventral striatum. So, the functions of the lateral orbitofrontal cortex, the rostral cingulate cortex, the amygdala, and the external pallidum would be affected, and impaired inhibition response, as well as impulse control, are expected to result in [11,12].

In the treatment of Parkinson’s disease, Levodopa proves to have higher efficacy. It enables better and more stable motility, but the adverse reaction stands in emphasizing psychiatric disorders like impulse control disorders (ICDs). The identification and recognition, in the early stages, of cognitive and behavioral problems complementary to PD patients’ symptoms are of high benefit for the management and efficient rehabilitative strategies. Even though it is not quite a high percentage, it should be noticed that about 15% of the patients develop relevant impulse control disorders (ICDs) caused by dopamine replacement therapy [7,9,12].

Impulse control disorders represent a group of psychiatric disorders defined mainly by a lack of control and characterized by an individual’s failure to control impulses that may be dangerous to oneself or to others. The lack of control is often distinguished by increased feelings of tension or anxiety before engaging in a behavior, a sense of pleasure, and a feeling of relief after committing the act [7,13].

There is a high risk for patients with Parkinson’s disease to develop single or multiple impulse control disorders (ICDs) [14]. The official and specific reports indicate the percentages of: (3.9–5.3%) for pathological gambling; (3.5–9.7%) for hypersexuality; (4.3–10.5%) for binge eating disorder; and (4.6–6.5%) for compulsive shopping [11]. A short description of the ICD aspects is provided next.

Pathological gambling (PG) is defined either as a habit or/and an impulse disorder. This disorder is chronic, and that is why it gets progressive, with evolution up to persistent and recurrent gambling behavior not able to be changed. PG has a significant negative impact on the family, social, and professional life [15].

Hypersexuality (HS) stands for compulsive behavior determined by an obsessive sexual set of mind towards sexual practice. In most cases, these are different from a person’s common previous sexual activities by focusing on media pornography or promiscuous sexual behaviors. HS would harm normal, ordinary life by overlapping tasks, duties, and objectives [16].

Binge eating disorder (BED) is the attitude of eating compulsively with no good feeling of having enough. The quantity of food one eats is much higher than that of ordinary people who eat in similar conditions. BS significantly impacts a person’s physics, health, and, not the least, social life [17].

Compulsive shopping (CS) represents the need to go shopping and buy lots of things, usually not really needed and requiring more money than one could afford. In most cases, this disorder generates the state of getting debits with difficulty in return and, therefore, serious financial problems. CS evidences low self-control ability and, associated with the lack of money, determines the low mood of the person’s and, therefore, accentuates the disorders [18].

The World Health Organization (WHO) released the ICD-11 2024 annual update [19]. The codes for the aspects of impulse control disorders studied in this article are mentioned next.

− Pathological gambling (PG), code F63.0 in ICD-10—turned into gambling disorder (GD), code 6C50 in ICD-11;
− Hypersexuality (HS), code F52.8 in ICD-10—turned into compulsive sexual behavior disorder (CSBD), code 6C72 in ICD-11;
− Binge eating disorder (BED), code F50.81 in ICD-10—keeps to binge eating disorder (BED), code 6B82 in ICD-11;
− Compulsive shopping (CS), code F63.8 in ICD-10—turned into compulsive buying-shopping disorder (CBSD), code 6C7Y in ICD-11.

As one can notice, the ICDs highly impact a person’s behaviors and health in a negative way. The consequences might hide the symptoms of PD. Development of addiction-like behaviors may be caused by several risk factors, such as young age, addiction disorder history, family history of alcohol or substance abuse, high novelty seeking or impulsivity traits, psychiatric comorbidity, depression, and current cigarette smoking [7].

Different types of medications used in the early stages of PD show adverse effects, specifically motor complications in the patient’s lives. The study [20] points out the fact that non-ergot DAs and ropinirole as monotherapy have reduced the risk of patients’ dyskinesia. More of it, pramipexole therapy develops lower risks of wearing-off and on-off fluctuations in patients diagnosed with early-stage PD.

It seems that the risk for patients with Parkinson’s disease to develop ICDs does not get higher in the absence of dopamine replacement therapy. Management of these disorders usually stands in dose reduction or, if the case, in discontinuation of dopamine agonists. It has been noticed that modifications to dopamine replacement therapy, particularly dopamine receptor agonists, could be associated with the improvement of the ICD symptoms. Still, there has to be paid attention to the accentuation of motor symptoms and, not the least, to the dopamine agonist withdrawal syndrome [11,21].

As mentioned in [22], there is still the need for careful monitoring and caregiver involvement in the therapy with dopamine agonist discontinuation for impulse control disorder patients.

Some other researchers [23] point toward the fact that there are no statistically significant differences between Parkinson’s disease patients who do develop or do not develop impulse control disorders when age and sex are considered, not to mention the levodopa equivalent daily dose and dopamine agonists.

Because of the increased number of PD in patients of different ages, unfortunately not only old ones, there is high interest in therapy to diminish, as much as possible, the associated ICD symptoms. This article is focused on research obtained with different pharmacological treatments applied to patients with ICDs in Parkinson’s disease. The sample group for this study is made of 48 persons, out of which 32 are men. The results were evaluated using the HAM-A scores, and the data obtained were statistically analyzed. ICDs typically improve after dopaminergic agonist reduction. The scope of this research study is to determine the adequate pharmacological treatment scheme for impulse control disorders in Parkinson’s disease-diagnosed patients and, therefore, to follow correct prevention methods. The directions for further research development are also envisaged in the final part of this article.

## 2. Materials and Methods

Epidemiological data on Parkinson’s disease evidenced by the AntiParkinson Romanian Association [24] reveal a number of about 70,000 patients diagnosed with Parkinson’s disease. Still, there is the statement that the real number is much higher, but because of relatively limited material resources in hospitals, as well as poor information means for the population, most of the patients do ignore early Parkinson’s disease symptoms. They come to specialists for diagnosis in the advanced stage of this disease.

The study is focused on patients with Parkinson’s disease recruited from neurologists, family doctors, and psychiatrists’ listings from April 2016 to March 2023, both from state and private hospitals. The patients were invited to participate in our study examining the pharmacological treatment of ICDs in PD.

There were 365 patients who had been diagnosed with PD by a neurologist, according to clinical criteria. Inclusion criteria for this study were current treatment with dopaminergic agonists, DAs (Pramipexol, Roponirol, or Rotigotine) for at least 6 months, and at least two risk factors (for example, socio-economic status, history of psychiatric illness, smoking habits/alcohol use). Based on these criteria, there were only 142 participants eligible.

Our study aims to set an adequate pharmacological treatment plan for patients with Parkinson’s disease who developed symptoms related to impulse control. Another objective of this study is to identify common variables and risk factors that could be associated with the symptoms of ICDs.

The clinical experience of the authors was considered adequate background for the design of the instrument required in gathering the information on each patient. This was conducted using a chart review (see Table 1). The chart included information on age, gender, years since PD diagnosis, history of psychiatric illness, socio-economic status, marital status, smoking habits, and alcohol use. There is evidence that all the patients included in the study belong to the White/Caucasian race. This information was considered to be useful, as proved by the authors’ experience and, also mentioned by [25], lifestyle, social involvement in peaceful but active family life, and artistic activities represent positive factors for avoiding or diminishing ICD in patients with PD.

The patients were interviewed by psychiatrists using the Hamilton Rating Scale for Depression (HAM-D) and Hamilton Rating Scale for Anxiety (HAM-A), specific rating scales for supporting the rating and follow-up of ICDs, allowing the monitoring of changes in symptom severity over time. Each of these patients was diagnosed and further selected for the study based on the clinical criteria mentioned in the diagnostic and treatment guidelines valid in the clinics where the study was carried out. As previously mentioned, the ICD-10 and ICD-11 are the envisaged criteria, too.

Once the multiple-item questionnaires were completed, a study and interpretation of the results were performed to finally set the sample size for the study on pharmacological treatment of impulse control disorders.

Based on HAM test results for anxiety, there were only 48 eligible subjects (patients) for our study. This sample size is considered to be relevant enough (95% confidence interval for the normal distribution) as it exceeds the assumed recommended number of 30.

Based on medical procedures and on the authors’ expertise, the pharmacological treatment plan was adjusted by diminishing the daily dose of specific medication (levodopa, pramipexol, and ropinirol) and introducing escitalopram.

The obtained results were evaluated by HAM-A scores, and the data were processed by tools of applied statistics (descriptive statistics and regression analysis)—assisted by the Statistic Process Control (SOC) software and the Design of Experiments (DoE, JMP) software-free trial version.

Patients were routinely followed by a neurologist and psychiatrist for the next 2 years.

## 3. Results

The sample of 48 patients consisted of 32 men and 16 women. The patients’ mean age was 54 years (39–66). They were diagnosed with medium to severe ICDs: pathological gambling (PG), hypersexuality (HS), binge eating disorder (BED), and compulsive shopping (CS). A history of anxiety disorder or depression associated with smoking and alcohol abuse was found in 83% of the patients.

These aspects, correlated to the demographic and clinical features, are shown in Table 1 and Table 2, respectively.

From the data in Table 2, it can be noticed a relative connection between psychiatric illness and ICD type as follows:

- Depression could be considered a cause for pathological gambling (PG);
- Anxiety disorder could be the background for compulsive shopping (CS) and for binge eating disorder (BED);
- Behavior disorder could be a cause for hypersexuality (HS).

Based on the data shown in Table 2, it could be determined the prevalence of each ICD for the time of the observation mentioned in the study, with the percentages of compulsive shopping at 29%, pathological gambling at 49%, binge eating disorder at 12.5%, and hypersexuality at 12.5%.

An observation emerging from this study survey is that it refers to the relationship between patients’ age and impulse control disorder type. Specifically, PG and HS are more frequent for relatively young patients; CS is more relevant for older patients, and BED is present, no matter the age.

**Figure 1:**
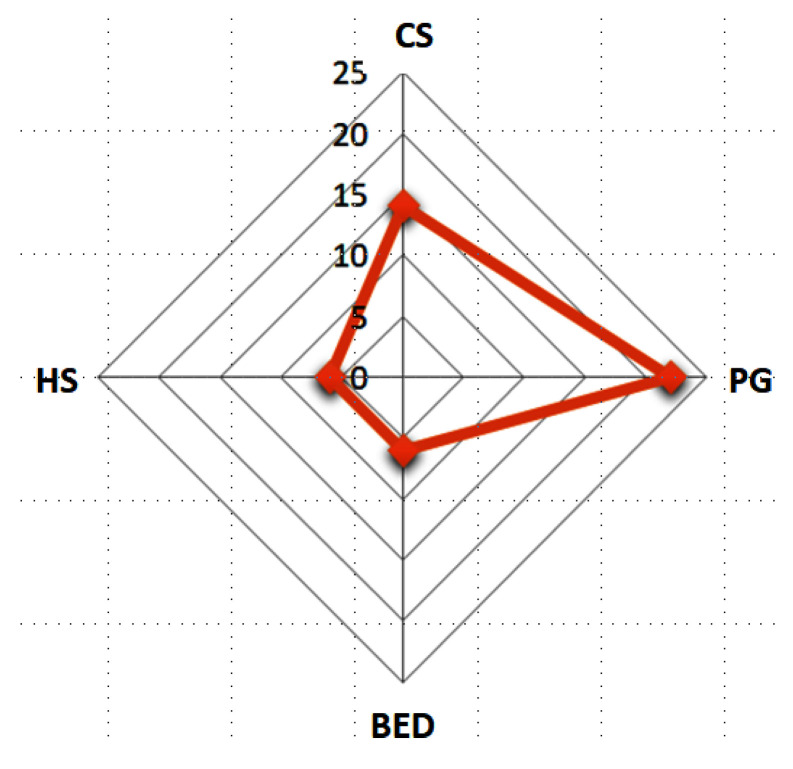
The ICD number of the sample patients.

A graphical representation of the number of each impulsive control disorder of the sample patients is evidenced in Figure 1.

Research on the dosage and type of treatment effect resulted in significant aspects as follows:

The management of ICDs in PD typically involves dose reduction. Therefore, the Levodopa dose was reduced to 500 mg daily, Pramipexol to 1.4 mg daily, or Ropinirole to 8 mg daily.

Patients received Escitalopram as monotherapy (10–20 mg daily) or in combination with Quetiapine (50–150 mg daily). Patients were divided into groups of treatment according to their addiction and severity.

Moderate forms of pathological gambling were treated with Escitalopram 10 mg daily in combination with Quetiapine 50 mg daily. Severe forms were treated with Escitalopram 20 mg and Quetiapine 150 mg daily.

Hypersexuality was treated with Escitalopram 10 mg and Quetiapine 100 mg daily; this targeted treatment from the authors’ perspective.

For binge eating disorder and compulsive shopping, we used monotherapy with Escitalopram 20 mg, equally divided (morning and evening).

**Figure 2:**
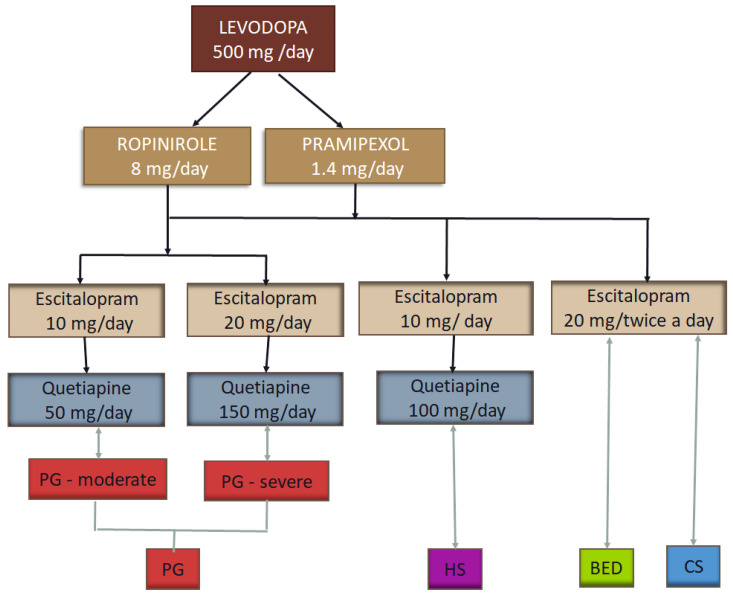
The tested pharmacological treatment for ICDs in Parkinson’s disease.

A schematic representation of the proposed pharmacological treatment is shown in Figure 2.

After 6 months of treatment, all patients showed a reduction in their impulse behavior, evaluated again by the Hamilton Rating Scale (HAM-A). Patients were also helped to avoid triggers by psychotherapy.

A significant improvement in their generalized anxiety disorder or depression symptoms was also found in earlier evaluations. Patients with BED and CS had a great reduction in their impulse behavior after 3 months. After 6 months, patients with HS rebuilt their family life and managed to return to daily activities without being affected by their behavior. Patients with PG noticed an improvement in their behavior and reported better control of their impulses.

**Figure 3:**
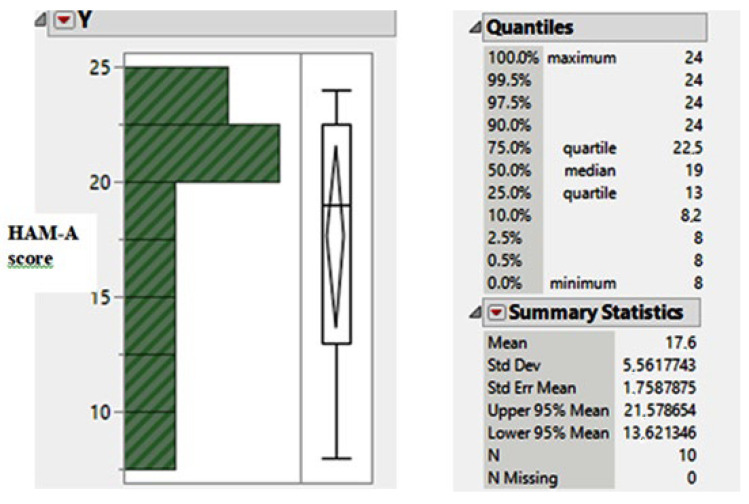
The descriptive statistics results for HAM-A scores.

A statistical analysis of this research study was conducted so as to determine the dependence relation between the pharmacological treatment dose and Hamilton Rating Scale, HAM-A, scores. The graphic plot and distribution parameter values of the scores—that prove the reduction in impulse behavior—are shown in Figure 3.

The mean value of 17.6 scores (for the HAM-A score) proves a moderate impulse behavior that, in fact, is an improvement of the severe ICDs evaluation before the pharmacological treatment previously mentioned (see also Figure 2).

**Figure 4:**
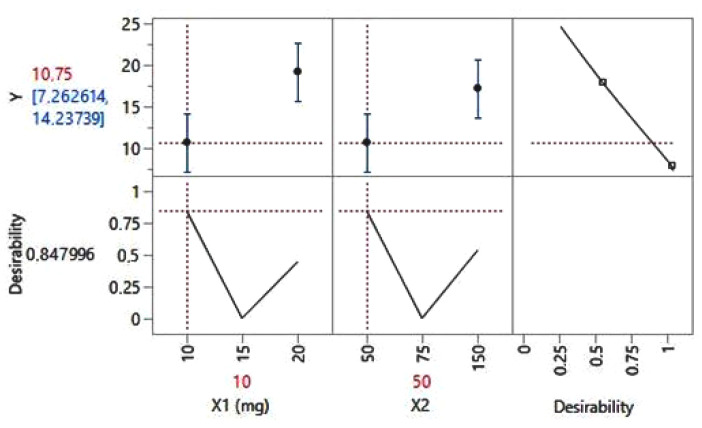
The prediction profiler for the HAM-A score.

By further running the statistical analysis of the data (Figure 4), there can be estimated a value of 10.75 HAM-A score for a daily dose of 10 mg of Escitalopram (code, ×1) in combination with Quetiapine 50 mg daily (code, ×2) (see Figure 4). The confidence interval proves limits in-between [7.26 and 14.23] or, in scale score, in-between [8 and 14]—that is, mild behavior disorder. For these statistical data, the coefficient of determination value (R squared) value resulted in 0.9713, thus proving the correct estimation of the pharmacological treatment plan.

As estimated previously in this study, ICDs typically improved after DA reduction, but there were a number of 8 patients (out of the 48 sample ones) who did not respond well to the reduced dose, and motor symptoms required compensatory increases of dopaminergic drugs. This is the case when, depending on each patient’s particular case, medication specific to another class has been applied.

These patients were further clinically surveyed from the point of view of tremor (frequency and amplitude) and psychiatric symptoms evolution through psychiatric, psychological, and psychometric reevaluations.

## 4. Conclusions

This article presents a study on the pharmacological treatment of impulse control disorders in Parkinson’s disease on a sample of 48 patients selected in hospitals. The authors have their clinical work. This study was carried out from April 2016 to March 2021 and is focused on different treatment plans. The results obtained were evaluated using the Hamilton Rating Scale, HAM-A.

The management of ICDs in PD typically involves dose reduction. Therefore, the Levodopa dose was reduced to 500 mg daily, Pramipexol to 1.4 mg daily, or Ropinirole to 8 mg daily.

Patients received Escitalopram as monotherapy (10–20 mg daily) or in combination with Quetiapine (50–150 mg daily). Patients were divided into groups of treatment according to their addiction and severity.

The relevant aspect is that the pharmacological treatment plan was adjusted by diminishing the daily dose of specific medication (levodopa, pramipexol, ropinirole) and introducing escitalopram. Treatment with Escitalopram ranging from 10 mg to 20 mg daily was useful in decreasing compulsive behaviors in patients with PD.

Besides the statistical analysis results obtained by the applied statistics tool for the data on HAM-A score, all results evidenced by this study are correlated to specific literature surveys—as evidenced by most of the cited references.

Further research development would be focused on the effectiveness and efficacy of Escitalopram treatment in improving ICDs in PD. Attention to the complementary action of family and social activities is also to be given.

We believe that AI could bring benefits regarding this research and could integrate information on the manifestations of Parkinson’s disease as well as the current treatment needed by any specialist in the field [26].

## Figures and Tables

**Table 1:** Sample patients’ demographic information.

Patient Code	Number of Patients	Age	Gender	Socio-Economic Status	Marital Status
1	4	42	Female	High income	Married
2	6	63	Male	Low income	Single
3	2	39	Female	Low income	Single
4	4	49	Male	Low income	Divorced
5	4	52	Male	High income	Married
6	6	52	Female	Average income	Married
7	6	66	Male	Retired	Married
8	4	63	Male	Average income	Widow
9	2	59	Male	High income	Single
10	4	50	Female	Unemployed	Married
11	2	61	Male	High income	Divorced
12	4	49	Male	Low income	Widow

All the patients in the study belong to the White/Caucasian race.

**Table 2:** Sample patients’ clinical information.

Patient Code	Period Since PD Diagnosis [years]	History of Psychiatric Illness	Smoking Habit/Alcohol Use	Medication	ICD	Hoehn and Yahr Score
1	>3	Anxiety disorder	Smoker	Pramipexol 2.1 mg daily	CS	3
2	>3	Depression	Smoker and heavy drinker	Levodopa 750 mg + Ropinirole 12 mg daily	PG	3
3	2	Anxiety disorder	None	Pramipexol 2.1 mg daily	BED	2
4	2	Anxiety disorder	Smoker and moderate drinker	Pramipexol 2.8 mg daily	BED	2
5	>3	Behavior disorder	Smoker and social drinker	Levodopa 750 mg + Pramipexol 2.1 mg daily	HS	3
6	>3	Anxiety disorder	Smoker	Levodopa 750 mg + Pramipexol 2.1 mg daily	CS	3
7	>3	Depression	Smoker and heavy drinker	Levodopa 750 mg + Ropinirole 16 mg daily	PG	3
8	>3	Depression	Smoker and heavy drinker	Levodopa 750 mg + Pramipexol 2.1 mg daily	PG	3
9	>3	Behavior disorder	Smoker	Levodopa 750 mg + Pramipexol 2.1 mg daily	HS	3
10	2	Anxiety disorder	None	Pramipexol 2.1 mg daily	CS	2
11	>3	Depression	Smoker and moderate drinker	Levodopa 750 mg + Pramipexol 2.1 mg daily	PG	3
12	>3	Depression	Smoker	Levodopa 750 mg + Pramipexol 2.8 mg daily	PG	3

CS—compulsive shopping; PG—pathological gambling; BED—binge eating disorder; HS—hypersexuality.

## Data Availability

Supplemental data are available upon reasonable request.
